# COVID-19 and Health Professions Education: A 360° View of the Impact of a Global Health Emergency

**DOI:** 10.15694/mep.2020.000148.1

**Published:** 2020-07-17

**Authors:** Adrian Rabe, Michael Sy, Wayne Yik Wai Cheung, Don Eliseo Lucero-Prisno

**Affiliations:** 1 Global Health Focus and Imperial College London; 2 National Teacher Training Center - Health Professions; 3 Global Health Focus; 4 London School of Hygiene and Tropical Medicine

**Keywords:** Global Health, curriculum, health emergency, COVID-19, technology

## Abstract

This article was migrated. The article was marked as recommended.

COVID-19 and the resulting lockdown policies have far ranging effects on health professions education (HPEd), with an impact in all its aspects, including curricula, teaching methods, student selection processes, educational outcomes, HPEd research, and student, teacher, and school welfare.

Adaptations to the pandemic and lockdown measures have leaned heavily on technology. The effects may have differences across various health professions particularly those with an emphasis on psychomotor skills. There may also be differences among populations of students, teachers and schools related to the availability, accessibility and cost of technology.

The rapid, forced change in the paradigms of health professions education across the globe may persist as long as lockdown measures and threat of resurgence remains, even as COVID-19 still sweeps across different countries.

## Background

COVID-19, a disease caused by the SARS novel coronavirus (SARS-nCoV-2), is a pandemic that has spread to nearly every country around the world ( WHO, 2020). The pandemic has a direct impact on health systems, with health care providers being overwhelmed in terms of the sheer capacity needed to care for patients, the lack of crucial diagnostic and therapeutic equipment and materials, and inadequate appropriate protective equipment to keep health workers safe (Institute for Fiscal Studies, 2020; Humphries, 2020; Benham *et al.*, 2020; Singhal *et al.*, 2020). As of 15 May 2020, there are more than 4.5 million SARS-nCoV-2 infections documented, with more than 300,000 mortalities (Our World in Data, 2020).

Countries have undertaken drastic measures to prevent the further spread of the disease, including highly restrictive lockdowns and selective allowances for mobility (World Economic Forum, 2020; Kaplan *et al.*, 2020). These policies vary per country but commonly provide a degree of freedom for designated “essential” workers and services ( Buchholz, 2020; Johns Hopkins University, 2020).

In line with these lockdown policies, many educational activities have since been suspended by universities globally, impacting even health professions education institutions (HPEIs) and potentially postponing or even preventing the entry of new professionals to the health workforce ( Arandjelovic, *et al.*, 2020). With the disease dynamic changing rapidly across different contexts, alongside our evolving understanding of the disease, it is not entirely known whether COVID-19 will disappear, or stay over the long term. Countries that have initially controlled the number of confirmed new cases are facing a resurgence of infections after loosening up their lockdowns ( Anthony, *et al.*, 2017; Li, *et al.*, 2020; Reuters, 2020; FRANCE24, 2020; Rogers, 2020). Thus, it is important to understand how health professions education (HPEd) is being impacted and what adaptations have to be considered in the near future and the long-term.

## Objective

The aim of this paper is to explore the impact of COVID-19 and the resulting lockdown on HPEd. Various aspects of HPEd are explored and potential solutions from literature collated and reported.

## Impact on the Curriculum

To contain the spread of COVID-19, most higher education institutions, including HPEIs, around the world were forced to close ( UNESCO, 2020). The impact however is particularly severe for institutions linked to hospitals that admit COVID-19 patients. These are frequently co-located within the HPEIs’ campuses. Moreover, students who are in their clinical placements and internships experience the most interrupted learning since, in most cases, their teaching-learning activities may not be readily translated to online platforms.

A recent mapping study discussed the responses of universities in 20 countries to the COVID-19 pandemic which included full suspension of all operations in the university, rapid curriculum redevelopment into online platforms, or allowing classes to continue while meeting minimum standards set by the government such as 1.5-meter social distancing or reduced social gatherings ( Crawford, *et al.*, 2020). These curricular changes, however, may not be favourable to some students especially those who do not have internet access at home or a private car to travel between home and school (since public transportation has been suspended). In the graduate school, some students are health professionals who may be redeployed to the frontlines and may not be able to work on their courses and assignments. While rapid curriculum redevelopment is necessary given the situation, it is important to put the students’ dispositions and considerations at the forefront of this process. However, in most health science curricula, the curriculum redevelopment process may compel faculty members to waive some curricular outcomes because the semester has been shortened or because the current situation does not allow attaining these outcomes.

In some countries, universities have collaborated with international organisations to temporarily divert their curricular offerings into COVID-related training sessions and webinars ( WHO, 2020). This diversion signposts to curriculum developers the need to include mandatory courses or electives on pandemic and emergency responses. The course can contain a range of topics or can be divided into different training sessions. We suggest the following topics that can be emphasised and integrated further into the curriculum of various health professions:

• Respiratory disease and infectious disease
• Home-based medical and health care
• Telemedicine
• Vaccination
• Community and population health
• Interprofessional education and collaborative practice

Patient contact is unique to the learning experiences of health science students and in health professions education. However, due to the restrictive measures caused by the COVID-19 pandemic, this may not be possible. For instance, in dentistry, aerosol-generating procedures have been prohibited hence these training opportunities may not be available for at least the next few semesters. Nursing and rehabilitation science students may have difficulty honing their patient care competencies since patient contact has been restricted and limited. While some faculty members have decided to increase the use of simulation-based education and online modules to facilitate clinical competencies, these options may not be readily available in resource-constrained universities. A recent study outlined the considerations for patient contact-based teaching and assessment that can be adapted in the health science curricula to prepare future generations of health professionals for future infectious disease outbreaks ( Arandjelovic, *et al.*, 2020).

## Impact on Teaching Methods

The rapid shift to digital pedagogy enabled creativity, innovation, and adaptability among students and health professions educators ( Sandars, *et al.*, 2020), but also increased stress and confusion among faculty members in health professions education. While online classrooms have had high-impact on student learning (Bao, 2020), this can only be possible with adequate support from faculty and full provision of technological and financial support from HPEI administration.

According to Thibault (2013), reforms in health professions education will require the increased use of new educational technologies. Therefore, teaching methods within curricula will need to shift to full online course offerings or blended learning at the very least. These new educational technologies (e.g., Moodle, Blackboard, Canvas, Google Classroom) include the use of health informatics, standardised patients, electronic patient records, and online modules.

With the increased use of online and remote approaches to facilitate learning, there is however a potential reduction of quality interaction when honing teamwork, communication, and interprofessional practice competencies. While these limitations are inevitable when using online platforms, group dynamics can still be effectively facilitated through early planning, having contingency plans, providing advanced readings, and creation of online rules. A short article from Nature ( Gewin, 2020) outlines tips for moving teaching online in the midst of COVID-19 pandemic including not converting entire lectures into video. In other words, if the lecture is normally given for three hours, the lecture can be summarized in a video format not longer than 30 minutes. Another tip is not relying solely on live video conferencing (e.g., Zoom Video Communications or Skype) since there are times when these applications can be overloaded and could eventually crash.

Teaching methods will need to have more diversity by providing both synchronous and asynchronous options. It is important that instructional designs are revised to provide advanced reading assignments, online and real-time learning sessions, self-study sessions, and additional resources in diverse formats (e.g., articles, videos, slides, e-books, e-libraries). Closely working with the college librarians will be useful in acquiring these resources while faculty members are staying at home.

Apart from the challenges in creating, maintaining, and improving remote and online learning ( UNESCO, 2020), it is crucial to maintain and develop professional behaviors when using online platforms. Particularly in health professions education, students are not only expected to attain clinical competencies but they are expected to integrate professional and ethical behaviors into those clinical competencies. In the midst of the rapid shift to online teaching, professors and even students still need to co-create expectations about proper decorum while attending online classes including punctuality, appearance, use of language, use of internet-originated language, emojis, and other rules. Furthermore, it is also important to train faculty members who are not acquainted with digital pedagogy to ensure that their students are not deprived of learning in the middle of COVID-19 pandemic.

## Impact on Student Selection

Processes of student selection have varied across different institutions prior to COVID-19 ( Salvatori, 2001). Many institutions employ multiple interviews in their processes ( Morris, 2009). Others include attitude, interpersonal skills, even manner of dress ( Danielsen, 2007, Wilcox and Lawson, 2018). With the COVID-19 pandemic, lockdown measures may prevent in-person interviews from taking place, likely replaced with online interviews and possibly with fewer steps in the process. These online interviews may eliminate some of the other cues that interviewers need for assessment (e.g. gestures, mannerisms, dress, social interaction, attitude), which necessitate an adjustment in the rubrics used to assess students in these interviews. There may even be room for the use of artificial intelligence to sift through applications, as has been done in job recruitment ( Wright and Atkinson, 2019; Mehrabad and Brojeny, 2007).

Moreover, many schools have chosen alternative methods for assessing students who plan to enter a health profession during COVID-19. Outcomes in secondary school or college may be altered due to mass promotion, online exams, and other adapted forms of assessment similar to the challenges seen in health professions educations ( Harvey, 2020, Baulkner and Sharfstein, 2020, Ferrer and Ryan, 2020) (see next section on assessment). These improvised assessments of students who are graduating from secondary school or college may not capture student suitability for health professions as previously assumed ( Cahn, 2015). This is more stark given the urgency and speed with which these adaptations were imposed, potentially not providing sufficient time for evidence-based, rigorously tested, and smooth transitions in assessment systems ( Wilcox and Lawson, 2018). Thus, the overall rubrics that consider these grades and outcomes to screen for the entry of students in the health professions have to be reviewed as well for appropriateness and validity.

Longer term issues arising from COVID-19 include changes in student motivation for entering health professions. Challenging conditions that health professionals have encountered along with the high risk of morbidity and mortality that comes as an occupational hazard, may shift perceptions of health professions ( Shanafelt, *et al.*, 2020, Huh, 2020). This may result in two potential effects: First, potential students whose perceptions were affected may shift instead into specialities and disciplines that have a lower risk during health emergencies ( Adams and Walls, 2020). Second, potential students may choose an entirely different career altogether, veering away from the health professions ( Razai, *et al.*, 2020).

Another important driver of student choice would be costs. The pandemic and resulting lockdown have impacted some job sectors more than others. Students of families whose incomes have been depleted by unemployment, salary cuts, and job shifts due to COVID-19 may choose low-cost educational options, or may even defer further education in favor of employment ( Modi, *et al.*, 2020).

These shifts in enrolment rates have started impacting HPEIs and universities whose intake season has commenced during the rise of COVID-19 in early to mid 2020. The longer term effects of these shifts in health worker shortages and maldistribution remain on the horizon as the pandemic continues ( Rose, 2020).

## Impact on Student Assessment

Many student assessment mechanisms across the health profession rely on an in-person examination, both written or oral ( LaDuca, *et al.*, 1984, Swanson, *et al.*, 1995, Greiner and Knebel, 2003). The premises of using these mechanisms include:

• Creation of standardized conditions for examination
• Cost-effective delivery of the assessment
• Relative assurance of recall and understanding (in assessments that don’t allow live access to references)
• Direct ability to evaluate and critique skills demonstrated by students (for assessments on skills)

Lockdowns due to COVID-19 have prevented these in-person examinations from occurring ( Lau, *et al.*, 2020). Instead, many schools have shifted to online assessments. These online assessments may still contain elements of an in-person examination, particularly written examinations. But even these may be impacted by the readily available references and learning material during the examination (skipping the “remember” cognitive skill in Bloom’s hierarchical learning Taxonomy), and the ability of students to game the system and potentially cheat (Fitzgerald, 2016, Huang, *et al.*, 2014). However, mechanisms to mitigate these may be implemented, such as timed display of questions and required input of answers and randomization of the order of questions and answer options ( Murad, *et al.*, 2010). Online assessments have even allowed HPEIs to explore multimedia types of presenting questions, now including audio and video, as well as new ways of introducing “real world” situations to these assessments ( Hew and Lo, 2018).

But more than an examination deployed at scale, other forms of assessment not requiring physical presence are being used. Assignments, concept mapping, and online focused group discussions may be considered in student assessment by HPEIs (Kaira and Kalra, 2020). HPEIs that have shifted to a problem-based or outcome-based learning model may have a particular advantage in these shifts, implementing a variety of assessment tools ( Murad, 2010).

The same adaptation might be more difficult for skills assessments. This area of assessment may suffer from the dual problem of a lack of opportunity for scrutiny of actual skill demonstration, and also the lack of exposure to learning material or even exclusion of some skills in the first place ( Fitzgerald, 2015, Simmons, *et al.*, 2010, Causby, *et al.*, 2014). COVID-19 has prevented advanced year HPEd students from getting adequate clinical exposure. This is crucial as internship and clinical clerkship allow for real world demonstration of concepts and skills learned in earlier years ( Rushforth, 2007). Examples of these skills include those with aerosolization (e.g., nebulization of medications, airway intubation, suction, dental extraction, etc) which presents an elevated risk of COVID-19 transmission. Moreover, in some HPEIs, students are fully withdrawn from hospitals and clinics, depriving them of much needed learning experiences ( Rose, 2020).

HPEIs have adopted a careful approach to this challenge ( Rose, 2020). Some HPEIs have allowed students to volunteer as consenting adults to continue training despite COVID-19 ( Huh, 2020). Others have allowed access to the hospital but only to non-COVID-19 wards.

Attitudes assessment will have varied impact due to the availability of assessment methods in HPEd ( Shaya and Gbarayor, 2006, Nawal, *et al.*, 2016, Danielsen and Cawley, 2007). Written forms of assessment may still proceed, such as through written case studies, essays and reaction papers. However, assessment of attitudes in the clinical setting may be hampered by the lack of opportunities for assessment (due to the urgency of the situation), as well as the lack of adequate opportunities to observe students in those settings.

A major challenge in student assessment would be national-level licensure examinations across the health professions ( Fitzgerald, 2015, Murad, 2010, Rose, 2020). In some countries, these are deployed mainly as written examinations held all at the same day at one site or at different testing centers (Cuff and Perez, 2017, Yudkowski, *et al.*, 2020). Countries where these examinations are not electronic may face the daunting task of having to create the infrastructure and logistical arrangements of transitioning into electronic examinations. These examinations tend to be less flexible and stricter in implementation, which may hamper the transition to online exams that regular student assessments can make.

Other countries with a multi-stage process of licensure (written, oral, and objective structured clinical examinations) may face more complicated logistical challenges in the deployment of these assessment methods (Cuff and Perez, 2017). Simulated patients in the real world may be replaced by virtual patients ( Amin, *et al.*, 2011, Kyaw, *et al.*, 2019). Students may have to perform examinations on models or fellow students with the assessor on teleconference. Some of these assessments will have to transition into a more traditional written examination as well.

## Impact on Educational Outcomes

Where countries choose to delay student assessments and national licensure examinations, there will be an immediate impact on educational outcomes, affecting the availability of the healthcare workforce and altering the array of opportunities available during and after the pandemic ( Yudkowski, *et al.*, 2020).

Perhaps an underappreciated aspect of assessment that may be better emphasized during the COVID-19 pandemic would be the ability to handle stress, the ability to adapt to rapidly evolving situations in resource-poor settings, management, and organizational skills ( David, *et al.*, 2005, Adams and Walls, 2020). These characteristics may now be more readily apparent in the pandemic and rubrics may be adjusted to include these elements in student assessment and professional licensure.

This aspect of change may further affect educational outcomes as a whole, altering the perspective of the health professions workforce that is being birthed and developed in the COVID-19 environment. This milieu of urgency, hazard, and on-the-fly innovation may produce emergency-ready health care workers who will face these situations in the future ( Kreitzer, *et al.*, 2009, Adams and Walls, 2020). Thus, the COVID-19-induced curricular changes discussed earlier will ultimately have an impact on the graduates of such curricula.

## Impact on Student, Teacher, and School Welfare

The COVID-19 pandemic poses not only physical health risks to the faculty members, professors, and university staff, but also risks to their mental health and other social determinants of health. Reports revealed some professors who have been infected with coronavirus have unfortunately died. Those who were quarantined and cannot resume teaching tasks may need substitution. However, in using online teaching platforms, teaching can be done asynchronously even with the absence of the teacher as underpinned by Activity Theory ( Westberry & Franken, 2015). On the other hand, emotional and mental health issues were also heightened among students and faculty members. The social isolation, economic pressures, and the potential impact of being trapped in abusive homes and environments may result in anxiety, depression, abuse, and violence ( UNESCO, 2020).

Moreover, adjunct and casual faculty members in private institutions are also at risk of losing their employment due to decreased funding following uncertainties in enrolment. Some universities in the Philippines have refunded tuition and matriculation to students when they shortened their semesters ( Magsambol, 2020), while some universities provided emergency stipend for either all students or selected students. Scholarship grants for overseas students have also been hampered due to restrictive measures on travels which consequently have raised issues on delayed scholarship provision. Some research funding has been redirected to higher priority research areas such as infectious diseases, emergency responses, vaccination, and telehealth protocols, consequently putting less priority to existing research in health professions education that are outside the current priority areas.

Health professions education programs are usually situated on campuses near training hospitals. This means that students, faculty, and staff working in the HPEd sector have a higher risk of getting infected by COVID-19 since they are more exposed in comparison to the non-health colleges or campuses. While this may not be applicable for all, there could be a possibility that health campuses may need to adapt to online teaching longer due to environmental constraints.

A wider impact may be seen in schools that have a larger proportion of intake from international students. These typically larger institutions may suffer from dropping enrollments and applications from other countries, along with the financial impact of loss of tuition fees, and the cultural impact from loss of diversity. Similarly, the shifts in trained teachers and fellows who travel to HPEIs to boost their research and teaching capacity will be impacted by COVID-19 and its associated travel restrictions.

## Impact on Health Professional Education Research

Research will always remain an integral part of academic work even if there are changes in learning environments and circumstances. Hurling the research environment into the epidemic quandary makes it even more interesting as the situation itself creates an interesting environment for research. In fact, the virus itself becomes the main topic of discussion resulting in many calls for COVID-related research and inquiries thus producing a massive output in a short period of time. This theme may predominate journal content over the course of the pandemic.

On the other side of this phenomenon, we have seen much more rapid review timescales in many journals. This pandemic may now shift expectations for the transition from study completion to publication. It is clear that shorter timelines are now possible than previously.

COVID-19 priority status for articles has also lifted the paywalls on many journals. These articles are frequently provided for free to enhance rapid dissemination of COVID-19 information across borders. The publication model and its financing system may certainly adopt such measures for health emergencies in the future.

All these changes may lead to better opportunities for research on HPEd’s process and content. Since the process of education has changed, there is change in the conduct of research as there is a shift from a normal and standard environment. There is also that possibility of stimulating new topics and more outputs in the context of a more focused and deliberate thought process in a forced lockdown. It is akin to a research sabbatical where the individual simply concentrates on the topic at hand to create higher quality ideas for research. The new environment may also lead to innovations discussed earlier, which can be a focus of HPEd research (e.g., Blackard, 2019).

Apart from the aforementioned research opportunities, COVID-19 has likewise brought challenges. Conducting research at home has difficulties particularly when, for many, their homes are not an ideal place for brainstorming. Even restrictions on physical activity and exercise may negatively impact creativity. Moreover, funding agencies may impose restrictions where one cannot procure materials or claim anything from the institution. The review process for proposals (funders, supervisors) may also not be optimal. Even if there are increased research outputs from postgraduate students, there may be no supervisors readily available. Research may also suffer as academics have to shift to online learning, resulting in more time and manpower focusing on the transition and on teaching itself. Academic interaction and networking, which helps generate collaborative research, is restricted due to cancellation of conferences or shifts to the virtual environment.

Thus, COVID-19 may provide opportunities but may also create impediments to research (e.g., Bakker & Wagner, 2020).

## Differential Impact on the Health Professions

With the likely HPEd impact of COVID-19 being focused on psychomotor outcomes in the curriculum, teaching strategies, student assessment and overall educational outcomes, professions which dwell more around skills face more challenges than those that focus around cognitive outcomes ( Nicholls, *et al.*, 2016, Gooding and Armstrong, 2016). These include programs for nursing assistants, respiratory therapy, anesthesiology, surgical specialties (general surgery, orthopedics, obstetrics and gynecology, ophthalmology), and professions requiring physical contact (physical therapy, occupational therapy). Conversely, mental health specialties may be less impacted as clinical assessments may be performed at a distance through teleconferencing.

An important exception to this potential impact would be psychomotor skills utilizing machines where these can be simulated or used in isolation. Examples of these professions include diagnostic radiology and pathology ( Kovacs, 1997).

However, as overall patient contact is decreased, even specialties requiring physical examination would be impacted. The limitations of video (resolution, lighting, color) may obscure details that would otherwise have been detected if the examination were done in person. Skills, such as auscultation and palpation would likely not be developed as well.

With psychomotor skills having less emphasis, HPEd can gear towards honing other essential competencies not given much attention in curricula. These include teaching, advocacy, research, creativity, collaborative working, and social accountability ( DePaola, 1994; Fatmi, 2013; Walker, *et al.*, 1998; Tolsgaard, 2015; Packard, *et al.*, 2012; Hojat, 2007; Williams, 2019; Adams and Walls, 2020).

## Differential Impact on Students and Teachers

With heavy reliance on internet connection to deliver education and enable academic interactions, this becomes a challenge when there are large disparities when accessing the same technology. The internet divide becomes more pronounced in the current situation when almost all educational institutions have to relocate to an online mode. As has been the experience in online global programs, asynchronous programs are being conducted not only because of the learners being located in different time zones of the world but also because of the internet connection where they can only access the technology in certain areas or certain periods during the day (e.g., Churchill, *et al.*, 2019). Thus, academic work and teaching has to be performed with those limitations.

In many online postgraduate programs (such as Masters in Public Health), many students work in remote areas and can only get back to their online material when they return to internet hubs. Sometimes, a way to address this is to do ‘cohorting’ where learners who come from the same zone can ‘meet’ at the same time for synchronous sessions. Another way of addressing the asynchronous issue is to record a session and keep the recording available for a certain period of time for those who are not able to access the session during the time period.

These new technologies used in online learning may not be embraced by everyone, especially “late adopters” ( Khan and Pred, 2001; Rogers, 2003). They include teachers, moderators and tutors who are not appreciative of this mode of teaching, particularly those who have predominantly used offline teaching methods prior to the pandemic. Furthermore, prior to COVID-19, it has always been theorized that online education is meant only for a certain group of learners thus one has a choice of whether to do it online or not. This theory may be tested in the current situation, with everyone being compelled to learn in this new kind of environment, resulting in learners who will be forced by necessity to embrace the approach (e.g., Reich, *et al.*, 2020). Other weaknesses of online learning are computer literacy (the digital divide), the skills of the facilitator, limitations of technology, and the curriculum ( Ramsetty & Adams, 2020), which vary across different countries and even within countries. There are also those who support online learning as it allows physically challenged learners and tutors to participate without physically being in a classroom.

## Take Home Messages

COVID-19 and the resulting lockdown policies have far ranging effects on health professions education (HPEd), with an impact in all its aspects, including curricula, teaching methods, student selection processes, educational outcomes, HPEd research, and student, teacher, and school welfare.

Adaptations to the pandemic and lockdown measures have leaned heavily on technology (see summary in Appendix 1). The effects may have differences across various health professions particularly those with an emphasis on psychomotor skills. There may also be differences among populations of students, teachers and schools related to the availability, accessibility and cost of technology.

The rapid, forced change in the paradigms of health professions education across the globe may persist as long as lockdown measures and threat of resurgence remains, even as COVID-19 still sweeps across different countries.

## Notes On Contributors

**Dr Adrian Paul J. Rabe** works as an honorary research fellow at Imperial College London, conducting teaching and learning activities as well as modelling the COVID-19 pandemic. He is also managing director of Global Health Focus, an international volunteer organization that counts as one of its strategies the deployment of health professions education programs in developing countries.

**Dr Michael P. Sy** is a Filipino occupational therapist and is currently an Associate Professor at the University of the Philippines Manila - National Teachers Training Center for the Health Professions. Apart from his research and works in occupational science, social justice, health professions education, and interprofessional education, he has been actively teaching courses in instructional design, teaching skills, and health professions evaluation.

**Wayne Cheung** is Director of Operations and Development at Global Health Focus, an international volunteer organization that counts as one of its strategies the deployment of health professions education programs in developing countries.

**Dr Don Eliseo Lucero-Prisno** is a Research Associate of the Global Health Institute of Wuhan University. He is the Managing Editor of the Wuhan-based international journal, Global Health Research and Policy. He is also a Senior Lecturer of International Health at the University of the Philippines Open University and a Tutor in Economics of Global Health Policy at the London School of Hygiene and Tropical Medicine. He recently was an Associate Professor of Public Health at Xi’an Jiaotong-Liverpool University (China) and the University of Liverpool (UK) for a decade. He is the Country Director for the Philippines of ACCESS Health International, a global think-tank in health and development based in New York.

## Appendices

### Appendix 1. Summary of Impact

**Table:**

Aspect of HPEd	Potential Impact
Curriculum	• Remodeling of curriculum (to include COVID- related topics) • Diverting of curricular offerings into COVID-related training sessions and webinars for international organisations • Limitations on patient-centric contact training • Considerations of adapting patient contact-based teaching to prepare future generations for infectious disease outbreaks
Teaching Methods	• Increased burden to faculty in supporting students technologically and financially • Shifts to new educational technologies (e.g. Moodle, Blackboard, Canvas, Google Classroom) • Overreliance and overloading the use of live video conferencing (e.g. Zoom Video Communications or Skype) which may lead to system crashes • The necessity to co-create expectations of online etiquette
Student Selection	• Improvisation of secondary/college examination methods, leading to misaligned student suitability for health professions • Decreased student pool for disciplines with higher risks in health emergencies • Completely new career choices for those previously intended for health professions
Student Assessment	• Reduced clinical exposure (Aerosolization) • Limited opportunity to assess students’ attitude in a clinical setting • Requirement of students to volunteer as consenting adults to continue training (hence assessed in clinical environments) amidst a pandemic
Educational Outcomes	• Delays in national licensure examinations, hence availability of healthcare workforce and opportunities before and after pandemic • Stress and resource-restrictive conditions related to COVID-19 to be taken into account when assessing students
Student, Teacher, and School Welfare	• Anxiety, depression, and violence for those isolating in abusive homes • Teacher unemployment due to enrolment uncertainties • Redistribution of research funding towards higher priority research areas such as infectious diseases, emergency responses etc (therefore limiting other areas)
HPEd Research	• Overwhelming research output on COVID-19 itself, eclipsing other journal content • Highly focused research due to lockdown conditions may bring HPEd into newly undiscovered domains • Liberalization of journal paywalls • Lower quality research as home environments are not ideal for brainstorming • Limitations on physical activity adversely affecting creativity • Collaborative research may become limited due to lack of networking and interaction from conference cancellations
