# Supplementary material for: COVID-19 and Health Professions Education: A 360° View of the Impact of a Global Health Emergency
Source: MedEdPublish (2016). 2020 Jul 17;9:148. [Version 1] doi: 10.15694/mep.2020.000148.1 (PMC10702676; doi:10.15694/mep.2020.000148.1)
Supplement: Peer review history

# Reviewer report: Reviewer response for version 1

**DOI:** 10.21956/mep.20095.r30960

**Published:** 2020-10-09

**Authors:** Barbara Jennings

**Affiliations:** 1 Norwich Medical School

This review has been migrated. The reviewer awarded 5 stars out of 5 Thank you for a really thought provoking and wide-ranging review. I will certainly be recommending this article to colleagues from many healthcare and medical education backgrounds who are involved with curriculum and workforce planning. Your appraisal of the adaptations needed and challenges for Teaching Methods resonated with me – working with instructional designers is critically important just now and so is thinking about the right balance for synchronous and asynchronous delivery – the over reliance on live videoconferencing is a ubiquitous problem & will probably only improve when staff experiment with and study technology enhanced learning and are supported by their organisations to do so. The impact summary and bibliography in the article will be valuable to many academics in clinical faculties. The covid-19 pandemic is far from over and we need to look for inclusive and robust methods to continue effective academic work (with the welfare of all staff & learners in mind) over this academic year and beyond.

Reviewer Expertise:

NA

No decision status is available

# Reviewer report: Reviewer response for version 1

**DOI:** 10.21956/mep.20095.r30962

**Published:** 2020-07-21

**Authors:** Jessica Servey

**Affiliations:** 1 Uniformed Services University of the Health Sciences F. Edward Hebert School of Medicine

This review has been migrated. The reviewer awarded 4 stars out of 5 Thank you. I think this is organized to consider multiple aspects of the crises and from multiple perspectives in the educational environment.

Reviewer Expertise:

NA

No decision status is available

# Reviewer report: Reviewer response for version 1

**DOI:** 10.21956/mep.20095.r30961

**Published:** 2020-07-18

**Authors:** John Cookson

**Affiliations:** 1 University of Worcester

This review has been migrated. The reviewer awarded 5 stars out of 5 Thank you; a very useful review of large amount of material, which otherwise becomes overwhelming

Reviewer Expertise:

NA

No decision status is available
